# Supplementary material for: Saccharomyces boulardii (CNCM I-745) improves intestinal damage in sepsis by remodeling intestinal flora structure
Source: Front Cell Infect Microbiol. 2025 Jul 31;15:1602792. doi: 10.3389/fcimb.2025.1602792 (PMC12350329; doi:10.3389/fcimb.2025.1602792)
Supplement: Supplementary file 1 [file DataSheet1.zip › summary/summary/8_Advanced_analyse/2_PICRUSt2_result/KEGG/Group.BW4_vs_AW4/PICRUSt2_KO_Group.BW4_vs_AW4_diff.pdf]

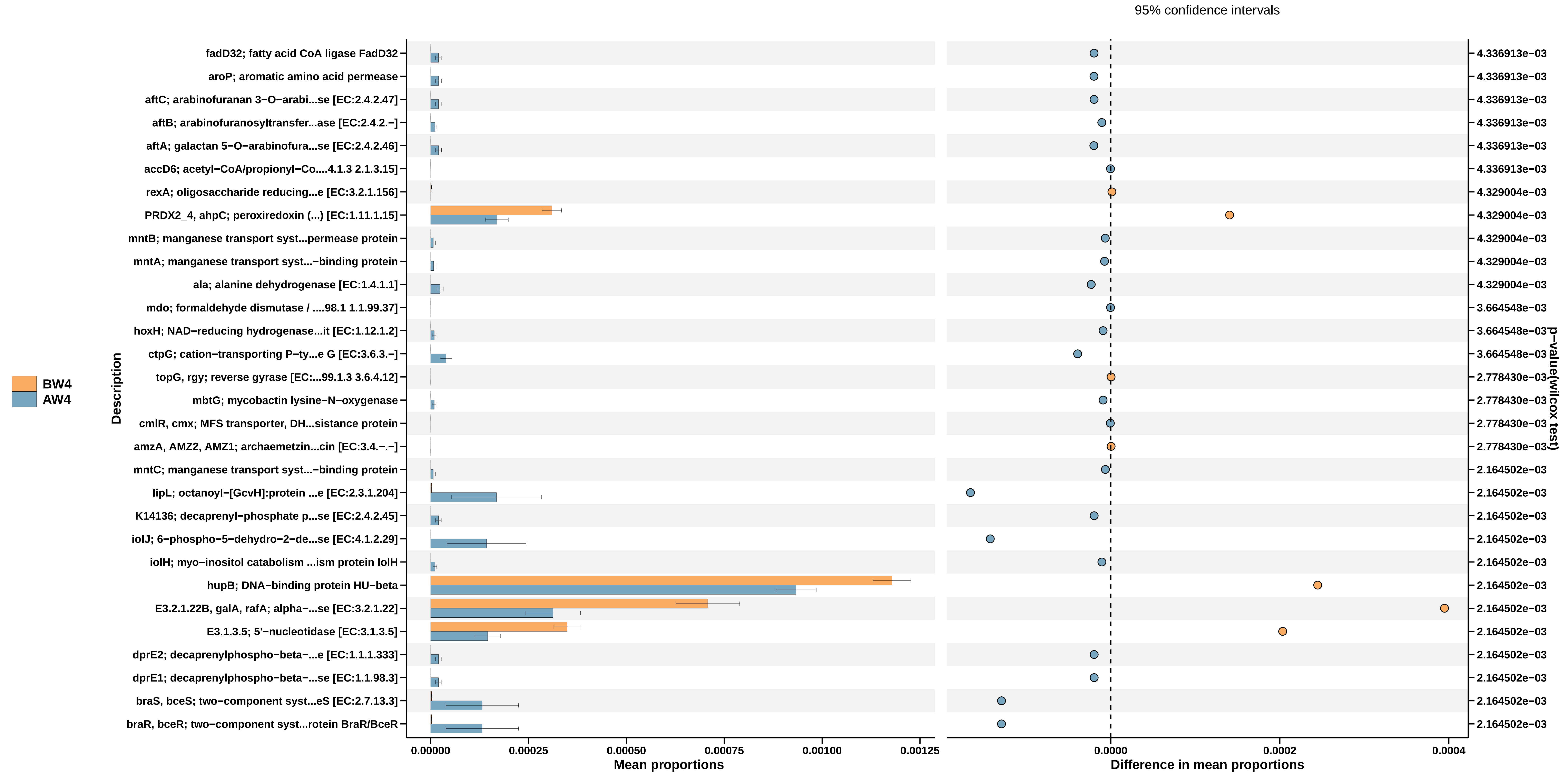
95% confidence intervals
fadD32; fatty acid CoA ligase FadD32
aroP; aromatic amino acid permease
aftC; arabinofuranan 3−O−arabi...se [EC:2.4.2.47]
aftB; arabinofuranosyltransfer...ase [EC:2.4.2.−]
aftA; galactan 5−O−arabinofura...se [EC:2.4.2.46]
accD6; acetyl−CoA/propionyl−Co....4.1.3 2.1.3.15]
rexA; oligosaccharide reducing...e [EC:3.2.1.156]
PRDX2_4, ahpC; peroxiredoxin (...) [EC:1.11.1.15]
mntB; manganese transport syst...permease protein
mntA; manganese transport syst...−binding protein
ala; alanine dehydrogenase [EC:1.4.1.1]
mdo; formaldehyde dismutase / ....98.1 1.1.99.37]
hoxH; NAD−reducing hydrogenase...it [EC:1.12.1.2]
ctpG; cation−transporting P−ty...e G [EC:3.6.3.−]
topG, rgy; reverse gyrase [EC:...99.1.3 3.6.4.12]
mbtG; mycobactin lysine−N−oxygenase
cmlR, cmx; MFS transporter, DH...sistance protein
amzA, AMZ2, AMZ1; archaemetzin...cin [EC:3.4.−.−]
mntC; manganese transport syst...−binding protein
lipL; octanoyl−[GcvH]:protein ...e [EC:2.3.1.204]
K14136; decaprenyl−phosphate p...se [EC:2.4.2.45]
iolJ; 6−phospho−5−dehydro−2−de...se [EC:4.1.2.29]
iolH; myo−inositol catabolism ...ism protein IolH
hupB; DNA−binding protein HU−beta
E3.2.1.22B, galA, rafA; alpha−...se [EC:3.2.1.22]
E3.1.3.5; 5'−nucleotidase [EC:3.1.3.5]
dprE2; decaprenylphospho−beta−...e [EC:1.1.1.333]
dprE1; decaprenylphospho−beta−...se [EC:1.1.98.3]
braS, bceS; two−component syst...eS [EC:2.7.13.3]
braR, bceR; two−component syst...rotein BraR/BceR
Description
BW4
AW4
0.00000
0.00025
0.00050
0.00075
0.00100
0.00125
Mean proportions
0.0000
0.0002
0.0004
Difference in mean proportions
4.336913e−03
4.336913e−03
4.336913e−03
4.336913e−03
4.336913e−03
4.336913e−03
4.329004e−03
4.329004e−03
4.329004e−03
4.329004e−03
4.329004e−03
3.664548e−03
3.664548e−03
3.664548e−03
2.778430e−03
2.778430e−03
2.778430e−03
2.778430e−03
2.164502e−03
2.164502e−03
2.164502e−03
2.164502e−03
2.164502e−03
2.164502e−03
2.164502e−03
2.164502e−03
2.164502e−03
2.164502e−03
2.164502e−03
2.164502e−03
p−value(wilcox test)
